# An Optimization-Based Technology Applied for Face Skin Symptom Detection

**DOI:** 10.3390/healthcare10122396

**Published:** 2022-11-29

**Authors:** Yuan-Hsun Liao, Po-Chun Chang, Chun-Cheng Wang, Hsiao-Hui Li

**Affiliations:** 1 Department of Computer Science, Tunghai University, Taichung 407224, Taiwan; 2 Bachelor’s Degree Program in Chain Store Management, Tainan University of Technology, Tainan 710302, Taiwan

**Keywords:** facial symptom detection, skin condition, YOLOv3, YOLOv4, Mask R-CNN

## Abstract

Face recognition segmentation is very important for symptom detection, especially in the case of complex image backgrounds or noise. The complexity of the photo background, the clarity of the facial expressions, or the interference of other people’s faces can increase the difficulty of detection. Therefore, in this paper, we have proposed a method to combine mask region-based convolutional neural networks (Mask R-CNN) with you only look once version 4 (YOLOv4) to identify facial symptoms by this new method. We use the face image dataset from the public image databases DermNet and Freepic as the training source for the model. Face segmentation was first applied with Mask R-CNN. Then the images were imported into ResNet-101, and the facial features were fused with region of interest (RoI) in the feature pyramid networks (FPN) structures. After removing the non-face features and noise, the face region has been accurately obtained. Next, the recognized face area and RoI data were used to identify facial symptoms (acne, freckle, and wrinkles) with YOLOv4. Finally, we use Mask R-CNN, and you only look once version 3 (YOLOv3) and YOLOv4 are matched to perform the performance analysis. Although, the facial images with symptoms are relatively few. We still use a limited amount of data to train the model. The experimental results show that our proposed method still achieves 57.73%, 60.38%, and 59.75% of mean average precision (mAP) for different amounts of data. Compared with other methods, the mAP was more than about 3%. Consequently, using the method proposed in this paper, facial symptoms can be effectively and accurately identified.

## 1. Introduction

It is human nature to love beauty. With the rapid development of technology and the economy, consumers are paying ever greater attention to skin care products, especially facial care. Skin care products have transformed from luxury items to indispensable necessities in daily life. According to a report by Grand View Research, Inc. published in March 2022, the global skin care market was worth US$130.5 billion in 2021 and is expected to grow at a compound annual growth rate (CAGR) of 4.6% from 2022 to 2030 [1].

With the prevalence of coronavirus disease 2019 (COVID-19) in the last few years, it has changed the operating model of many companies and consumer buying behavior [2]. Many consumers switched to online purchases instead of physical channels. According to a report by Euromonitor International, e-commerce will expand by another $1.4 trillion by 2025, accounting for half of global retail growth [3]. As consumption patterns change, numerous brands have begun to use artificial intelligence (AI), augmented reality, virtual reality, and other technologies to serve their customers.

In the past, consumers in the physical channel often relied on the advice of salespeople in making product purchases, but when online shopping is conducted, consumers can only make product selections according to their own preferences. Since everyone’s skin condition is different, some consumers with sensitive skin may experience allergic reactions after using products unsuited to them [4]. According to a survey report, 50.6% of 425 participants had experienced at least one adverse reaction to product use in the past two years, experiencing conditions including skin redness (19%), pimples (15%), and itching (13%), and 25% of these participants had problems caused by the use of unsuited skin care products [5]. Thus, the use of unsuitable skin care products can not only seriously harm consumers’ skin but also have a terrible impact on a manufacturer’s reputation [6].

On the other hand, as the COVID-19 epidemic has swept the world in recent years, people will wear masks whenever they go out [7,8]. Because of that, people wear masks for a long time every day, and the problems with facial skin are increasing daily [9,10]. In particular, the proportion of medical staff who have facial skin problems has greatly increased, among which contact dermatitis, acne and pimples, and rosacea are the most common [11,12,13]. In order to help people take care of their own facial health while cooperating with the epidemic prevention policy, we hope this research can offer cogent advice on skin care issues.

In the past, it was not easy to create an intelligent skin care recommendation platform due to the limitations imposed by image processing techniques [14,15]. With the vigorous development of deep learning in recent years, image-processing techniques have become more mature. There is a thus glimmer of light on this issue. There are three common facial skin problems, which are acne, spots, and wrinkles [16,17,18]. We chose these three as the feature categories for this study.

The first type, acne, is caused by abnormal keratinization of pores and strong secretion of sebaceous glands, resulting in excessive oil that cannot be discharged and blocked hair follicles. The main reasons are insufficient facial cleansing, endocrine disorders, and improper use of cosmetics and skin care products [19,20].

The second type, freckles, is mainly caused by excessive sun exposure. When the skin’s melanocytes are overstimulated by ultraviolet light, it causes the cells to produce more melanin, which in turn causes freckles. Other causes include endocrine disorders and bodily aging [21,22,23].

For the third type, wrinkles, the most common cause is dryness. Dry lines often appear on the cheeks and around the corners of the mouth. Due to the lack of water in the skin, the outermost sebum film cannot play a protective role, the moisturizing ingredients (ceramides) under the stratum corneum are reduced, and the skin cannot retain water and begins to shrink and sag. Moisturizing as soon as you notice fine lines will most likely eliminate them [24,25,26].

Face recognition segmentation is very important for symptom detection, especially in the case of complex image backgrounds or noise. The complexity of the photo background, the clarity of the facial expressions, or the interference of other people’s faces can increase the difficulty of detection. In the past, Adjabi et al. [27] pointed out that the two-dimensional face recognition methods have holistic methods, local (geometrical) methods, local texture descriptors-based methods, and deep learning-based methods. Among them, the deep learning method is the current development direction, so in our face recognition method, we selected the deep learning algorithm Mask R-CNN [28,29,30,31].

Mask R-CNN is an instance segmentation algorithm. It can identify each object instance for every known object within an image. Because of that, we use it to detect where a face is and turn the region that Mask R-CNN was not predicted to black. After detecting the region of the face, YOLOv3 [32,33,34,35,36,37,38,39,40,41] and YOLOv4 [11,42,43] are deployed to detect facial symptoms. The reason for choosing this method in order to solve the problem is that pictures of facial lesions are not easy to obtain. The study results show our proposed method is effective in improving recognition rates. Although the facial images with symptoms are relatively few, we still use a limited amount of data to train the model. The experimental results show that our proposed method still achieves 57.73%, 60.38%, and 59.75% of mean average precision (mAP) for different amounts of data. Compared with other methods, the mAP was more than about 3%. Consequently, using the method proposed in this paper, facial symptoms can be effectively and accurately identified.

The organization of this paper is as follows. In Section 2, this paper describes the related work of Mask R-CNN, YOLOv3, and YOLOv4. In Section 3, the materials and our method are described. Next, we discuss the experimental results. Finally, we provide our results, discussion, and future work. The detailed abbreviations and definitions used in the paper are listed in Table 1.

## 2. Related Work

**Figure 1:**
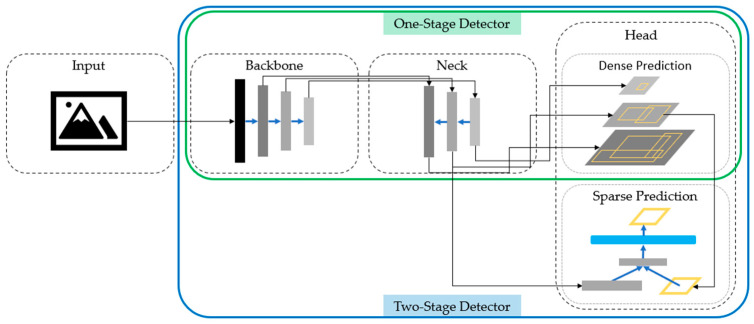
Object detector architecture.

Object Detection is an important aspect of image recognition. Many results have been reported in object recognition, vehicle recognition, person recognition, and face recognition. The Object detector model applied to object detection consists of four parts: Input, Backbone, Neck, and Head, as shown in Figure 1. The Backbone part is usually composed of a trained neural network that aims to capture basic features to improve the performance of target detection. The neck part is used to extract different feature maps at different stages of the backbone. The last part of the Head can be divided into Dense Prediction (one-stage) and Sparse Prediction (two-stage).

There are several common two-dimensional face recognition methods: holistic methods, local (geometric) methods, methods based on local texture de-labeling, and methods based on deep learning [27]. Deep learning methods are the current trend. To improve face skin symptom detection, we conduct a deep learning review to introduce Mask R-CNN, YOLOv3, and YOLOv4.

### 2.1. Mask R-CNN

**Figure 2:**
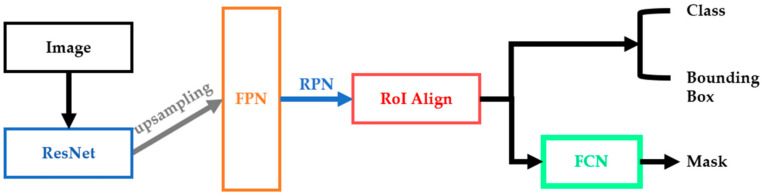
The structure of Mask R-CNN.

The two-stage model of Mask R-CNN combines the two-stage model of Faster Region-based Convolutional Neural Networks (Faster R-CNN) [44], and the Feature Pyramid Networks (FPN) [45] method uses feature maps with high feature levels in different dimensions for prediction as Figure 2.

It also improves the shortcomings of Region of Interest (RoI) pooling in Faster R-CNN so that the longitude of the bounding box and object positioning can truly reach the pixel level, increasing the accuracy rate by 10~50%.

Mask R-CNN consists of:

- Backbone: ResNet-101 [46];
- Neck: FPN [45];
- Head: Dense Prediction(one-stage): RPN [44]

Sparse Prediction(two-stage): Mask R-CNN [28].

There have been many previous studies using Mask R-CNN. Zhang et al. [47] created a publicly available large-scale benchmark underwater video dataset to retrain the Mask R-CNN deep model, which in turn was applied to the detection and classification of underwater creatures via random under-sampling (RUS), achieving a mean Average Precision (mAP) of 62.8%. Tanoglidis et al. [48] use Mask R-CNN to solve the problem of finding and masking ghosts and scattered-light artifacts in DECam astronomical images.

### 2.2. YOLOv3 

The YOLOv3 [49] detector was developed to ensure symptoms detection would be more objective. The backbone of YOLOv3 is Darknet-53 which is more powerful than Darknet-19. The neck part includes FPN [45], which aggregates the deep feature maps of DarkNet-53.

YOLOv3 consists of:

- Backbone: Darknet-53 [49];
- Neck: FPN [45];
- Head: YOLO [50].

In the field of YOLOv3, Khan et al. [51] used this method and Microsoft Azure face API to perform face detection and face recognition, respectively, with a real-time automatic attendance system for face recognition, and this system enjoys a high accuracy rate in most cases. Menon et al. [52] implemented face recognition using both R-CNN and YOLOv3 algorithms. Compared with other algorithms, it has a higher processing speed.

### 2.3. YOLOv4

YOLOv4 improves various parts of YOLOv3, including the Backbone, Neck, and Head. Not only does it build an efficient and powerful object detection model that can be trained using a 1080Ti or 2080Ti GPU, but it also verifies the influence of the Bag-of-Freebies and Bag-of-Specials target detection methods of State of the Art (SOTA) and improves some tricks and SOTA methods, making it more efficient, and able to train on a single GPU.

YOLOv4 consists of the following:

- Backbone: CSPDarknt-53 [53];
- Neck: SPP [54], PAN [55];
- Head: YOLOv3 [49].

Prasetyo, Suciati, and Fatichah [56] discussed the application of YOLOv4-tiny to the identification of fish body parts. Since the author of this article found that the accuracy of identifying specific parts of fish is relatively low, the author Modified Yolov4-tiny using wing convolutional layer (WCL), tiny spatial pyramid pooling (Tiny-SPP), bottleneck and expansion convolution (BEC), and additional third-scale detectors. Kumar et al. [57] used tiny YOLOv4-SPP to achieve better performance in mask detection than the original tiny YOLOv4, tiny YOLOv3, etc., and the mAP reached 64.31%. Zhang et al. [58] found that compared with YOLOv4, the proposed weight Improved YOLOv4 has a 3.45% increase in mAP, while the weight size is only 15.53% of the baseline model, and the number of parameters is only 15.84% of the baseline model.

## 3. Materials and Methods

**Figure 3:**
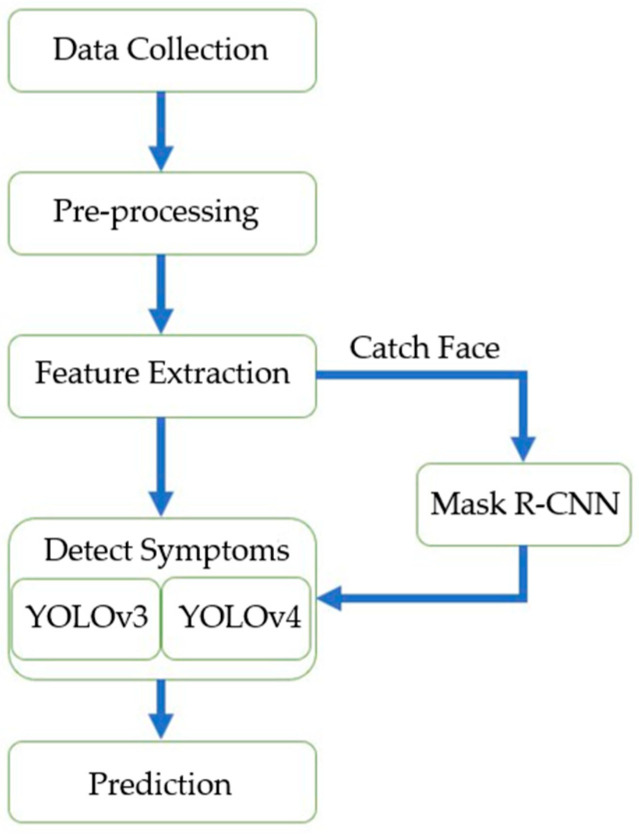
The overall schematic of the proposed model.

In this paper, our experimental procedure approaches showed in Figure 3, including data collection, pre-processing of the dataset, feature extraction, and training and testing of the target detection algorithm. The detailed procedure is as follows:

### 3.1. Data Collection

Data collection is divided into simple faces and pictures of faces with symptoms. A large amount of data can be obtained from the parts of the human face, but disease data is not easy to obtain. At present, we have collected about 1500 pieces of symptom data, all of which are taken from the public databases DermNet [59] and Freepic [60]. Because of that, this paper will focus on how to use a small amount of data to drive a more efficient model.

### 3.2. Pre-Processing

Before labeling the picture, we resize all of the pictures for a uniform length and width of 1000 × 750. One of the reasons is to avoid the need to mark the image too small, resulting in the area of the RectBox or polygons being too small when labeling it. The second is to avoid the problem that images of certain sizes cannot be read when using deep learning algorithms.

### 3.3. Feature Extraction

Symptoms are defined into 3 classes: named acne, freckles, and wrinkles. The main types of acne are Whitehead, Blackhead, Papule, Pustule, Nodule, and Cysts. We take Whitehead, Papule, Pustule, and Nodule as the acne characteristics of this study, as shown in Table 2.

The technical terms for freckles are Ephelides and Lentigines, and we define both as freckles for analysis, as defined in Table 3.

The last type, wrinkles, has a total of 12 kinds. We selected six horizontal forehead lines, Glabellar frown lines, Periodic lines, Nasolabial folds, Cheek lines, and Marionette lines as the characteristics of wrinkles in this paper, Table 4.

We used three different numbers of datasets to train the diagnosis of symptoms with splitting the training data into acne: 50%, freckles: 25%, and wrinkles: 25%, as shown in Table 5.

### 3.4. Detect Symptoms

**Figure 4:**
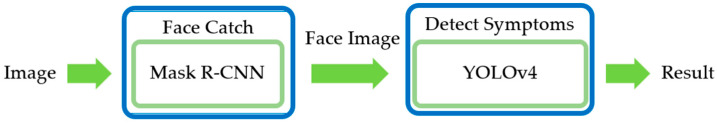
The structure of detection symptoms.

The methods proposed in this research are divided into those with face recognition and those without face recognition. For the face recognition algorithm, we use Mask R-CNN to maintain the primary color of the identified Region of Interest (RoI) and turn the uninteresting areas into black. Then we use YOLOv4 to identify the symptoms and further compare the accuracy of YOLOv4 in the identification of symptoms. The detection structure is shown in Figure 4.

#### 3.4.1. Face Detection Process

**Figure 5:**
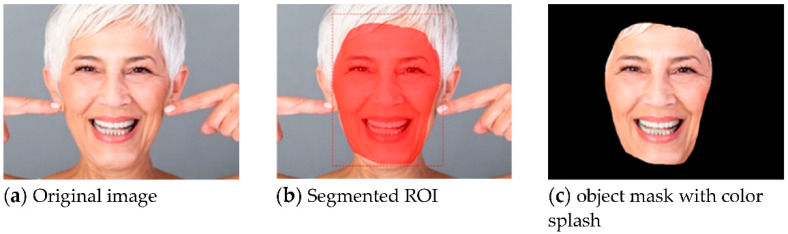
The results of face detection using Mask R-CNN.

The face recognition uses Mask R-CNN to keep the primary color of the RoI being recognized and transform the uninteresting regions into black. The detailed procedures are shown in Algorithm 1. The results of the face detection process are shown in Figure 5. Figure 5a shows the original image. After the Mask, R-CNN recognizes and marks the position of the face, as in Figure 5b. At last, the part outside the face area is transformed to black by using a color splash, as in Figure 5c.
**Algorithm 1** Face detection procedures**Input:** Original image 
**Output:** The image with only the face  1:Set the environment variables to match the features of the face.  2:Adjust the image to fit the requirements.  3:Pass the processed image into ResNet-101 and obtain the corresponding feature map.  4:FPN corrects the size of the RoIs in the feature map.  5:RPN classifies these RoIs and filters out the background, and BB regression corrects the BB of the RoI.  6:Use RoI alignment to split the remaining RoIs into facial RoIs and non-facial RoIs.  7:Use BB Regression to fix the BB of RoI again and generate the mask with FCN after classification.  8:Keep the facial part after the non-facial part becomes black.  9:End.

#### 3.4.2. Symptoms Detection Process

**Figure 6:**
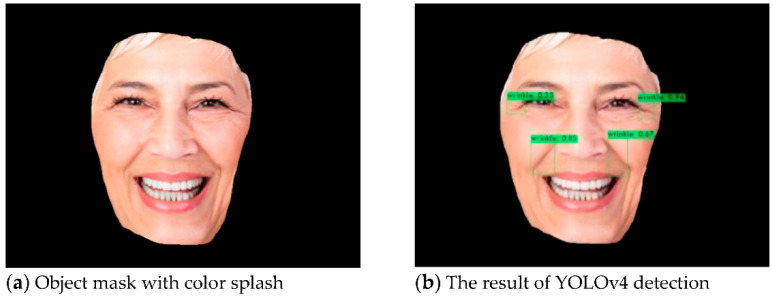
The result of symptoms detects using YOLOv4.

We used YOLOv4 to perform symptom identification and compared the accuracy of YOLOv4 further. The detailed procedures are shown in Algorithm 2. The results of the symptoms detection process are shown in Figure 6. We use the resulting image in the previous section of face recognition to identify skin symptoms, as in Figure 6a. YOLOv4 is used to identify and mark the position of acne, freckles, and wrinkles in the image, and the output will be the final result, as in Figure 6b.
**Algorithm 2** Symptoms detection procedures**Input:** Only face image **Output:** The symptom recognition  1:Using CSPDarknet53 transforms feature maps of different sizes in different convolutional layers.  2:Use SPP to transform feature maps of any size into feature vectors of fixed size to improve the perceptual field of view.  3:Use PAN to blend three feature maps of different sizes.  4:Pass the result of step 4 to the head of YOLO and output the three feature maps: (19,19,num_anchor*(num_classes + 5)), (38,38,num_anchor*(num_classes + 5)), (76,76,num_anchor*(num_classes + 5)).  5:Three different-size feature maps are used to calculate the prediction BB.  6:The IoU is compared with the ground-truth BB to calculate the IoU loss.  7:End.

### 3.5. Mean Average Precision for Evaluation

Mean average precision (mAP) or simply just referred to as Average Precision (AP), is a popular metric used to measure the performance of models. AP values are calculated over recall values from 0 to 1.

Traditional Intersection over union (*IoU*) = 0.5, *IoU* means the ratio of the area of overlap and area of union of the predicted bounding box (BB) and test data label bounding box.

(1) $$IoU=\frac{area\;of\;overlap}{area\;of\;union}$$

(2) $$Precision=\frac{TP}{TP+FP}=\frac{TP}{total\;positive\;results}$$

*TP*: True positive (*IoU* > 0.5 with the correct classification).

*FP*: False positive (*IoU* < 0.5 with the correct classification or duplicated BB).

(3) $$Recall=\frac{TP}{TP+FN}$$

*FN*: False negative (No detection at all or the predicted bounding box has an *IoU* > 0.5 but was the wrong classification).

The general definition for the Average Precision (*AP*) is finding the area under the precision-recall curve above.

(4) $$AP=\frac{1}{N}\sum_{Recall_{i}}^{\;}Precision\left(Recall_{i}\right)$$

*N*: the number of queries.

## 4. Results and Discussion

First, we used images collected from public databases on the internet for training, using YOLOv3 and YOLOv4 to train symptom recognition, respectively, and used Mask R-CNN to train face labels.

We compared the model trained by YOLOv3 and the model trained by YOLOv4, determined which feature identification can achieve better results, and also tested whether the number of images in the training set influences the training model.

For the training set of YOLOv3, we used the training set of 500, 1000, and 1500 to train and generate the results of Table 6. For the model of 1500, we obtained the average value of mAP and the model with the highest value in the YOLOv3 training set.

The training results of YOLOv4 are also in line with the conclusions we have drawn from YOLOv3. The training of 1500 images obtains a better model in the symptom labels, as in Table 7.

Among the training results of Mask R-CNN, the training set with the largest number of sheets is also the best in this study, which is also in line with the conclusions we have drawn from YOLOv4 and YOLOv3. The more training sets, the better the results. The training results are presented in Table 8.

According to the above three statistical charts, we use the best model in YOLOv3 and YOLOv4 to identify the pictures of symptoms with complex backgrounds in the picture set.

Then analyze whether YOLO’s symptom identification will be as we expected after the Mask R-CNN removes the parts other than the face in these image sets. It is better than the original YOLO to directly identify images with complex backgrounds. The accuracy of statistics is presented in Table 9.

From the experimental results, we can find that when the number of training sets is inconsistent, there will be different results. When the number of training sets is too small, it is more likely the trained model will have unstable recognition. In Table 9, we can find that the Mask R-CNN training data set is unstable at 56.53% of 100 images and 53.70% of 250 images but stable and improved at 58.13% of 500 images. Therefore, the experimental results in Table 9 can demonstrate our designed model adequately. We first use Mask R-CNN to remove the parts other than the face in these images. Then, we use YOLO to identify the symptoms of the face more effectively. Compared with YOLOv3 alone, the results were only 54.52%, 50.01%, and 55.68%. Using our method (Mask R-CNN + YOLOv3), the results are 55.02%, 52.39%, and 58.13%, which are at least a 1% mAP improvement.

With YOLOv4 alone, only 58.74%, 56.98%, and 56.29% were achieved. Using our method (Mask R-CNN + YOLOv4), the results are 57.73%, 60.38%, and 59.75%, which are at least a 3% mAP improvement. Therefore, our proposed method can effectively improve the results of facial symptom recognition for symptom pictures with complex backgrounds.

**Figure 7:**
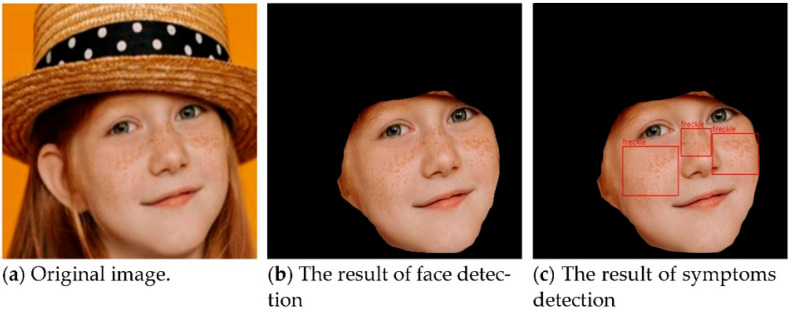
The results of detect symptoms process.

Therefore, the results of our method process as shown in Figure 7. First, we input the images that may have noise, as in Figure 7a. Then, we use Mask R-CNN to remove the parts other than the face, as in Figure 7b. Finally, symptom recognition can be predicted with YOLOv4, as in Figure 7c.

## 5. Conclusions

In this study, we compared YOLOv3, YOLOv4, Mask R-CNN + YOLOv3, and Mask R-CNN + YOLOv4 with the same number of diseases datasets and found that the accuracy of our method has improved significantly. At the same time, we experimented with Mask R-CNN before YOLO identified symptoms. The results indicate that our proposed method still achieves 57.73%, 60.38%, and 59.75% of mean average precision (mAP) for different amounts of data. Compared with only using YOLOv4 to symptom detect the image has noise, the mAP was more than about 3%.

Instead of relying on one image recognition algorithm for training, we combine multiple algorithms. We choose different algorithms in each stage according to the different features of the images. First, we segment the complex images to remove redundant images and noise. Then, we enhance the required image features for the detection of skin symptoms. This study reduces the difficulty of model training and model training time and increases the success rate of detailed feature identification.

In general, AI research requires large training data sets. However, in the field of “Face Skin Symptom Detection” research, there are problems from insufficient image data and uneasy acquisition. The proposed approach can be used to train a model with fewer data sets and time and has good identification results.

Under the influence of COVID-19, consumers’ shopping habits have changed. With so many skin care products available on the internet, choosing the right skin care product is an important issue. Through our research, consumers can understand their own skin symptoms to facilitate the proper selection of skin care products and avoid purchasing unsuitable products that may cause skin damage.

In the future, we will combine the results of our research on face skin symptom detection into a product recommendation system. The detection results will be used in a real product recommendation system. We expect to design an App for facial skincare product recommendations in the future.

## Figures and Tables

**Table 1:** List of abbreviations and acronyms used in the paper.

Abbreviation	Definition
AP	average precision
BB	bounding box
BEC	bottleneck and expansion convolution
Faster R-CNN	faster region-based convolutional neural networks
FCN	fully convolutional networks
FPN	feature pyramid networks
GPU	graphics processing unit
IoU	intersection over union
Mask R-CNN	mask region-based convolutional neural networks
PAN	path aggregation network
RoI	region of interest
RPN	region proposal network
RUS	random under-sampling
SOTA	state-of-the-art
SPP	spatial pyramid pooling
Tiny-SPP	tiny spatial pyramid pooling
WCL	wing convolutional layer
YOLO	you only look once
YOLOv3	you only look once version 3
YOLOv4	you only look once version 4

**Table 2:** Types of common acne in training sets [18].

Acne Type	Size	Color	Pus	Inflammatory	Comments	Image
White-head	Tiny	Whitish	No	No	A chronic whitehead is called milia	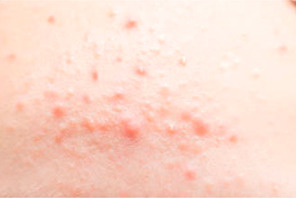
Papule	<5 mm	Pink	No	Yes	Very common	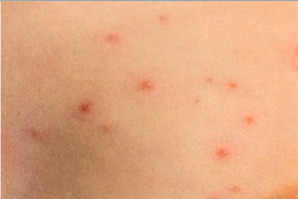
Pustule	<5 mm	Red at the base with a yellowish or a whitish center	Yes	Yes	Very common	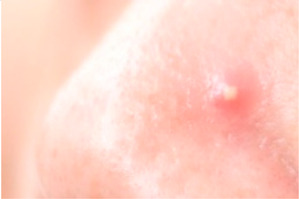
Nodule	5–10 mm	Pink and red	No	Yes	A nodule is similar to a papule but is less common	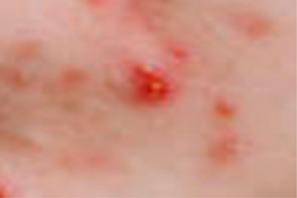

**Table 3:** Types of Face Wrinkle in training sets [20].

Freckle Type	Ephelides	Lentigines
Appearance	First visible at 2–3 years of age after sun exposure, partially disappears with age	Accumulate with age, common after 50, stable
Effects of sun	Fade during winter	Stable
Size	1–2 mm and above	mm-cm in diameter
Borders	Irregular, well defined	Well defined
Color	Red to light brown	Light yellow to dark brown
Skin type	Caucasians, Asians, skin type I-II.	Caucasians, Asians, skin type I–III
Etiology	Genetic	Environmental
Image	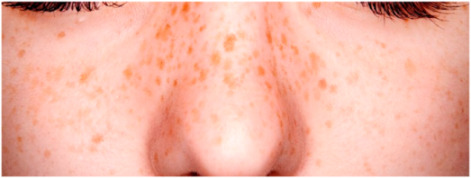	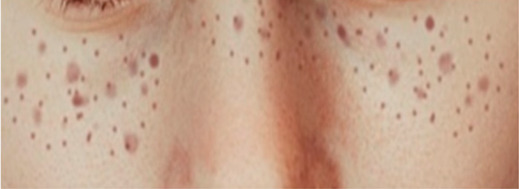

**Table 4:** Characteristics of ephelides and lentigines [23].

Wrinkle Type	Position	Image
Horizontal forehead lines	Forehead	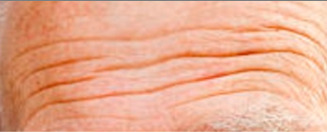
Glabellar frown lines	Between eyebrows	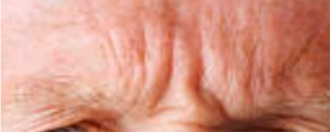
Periorbital lines	Canthus	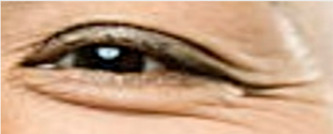
Nasolabial folds	Nose to mouth	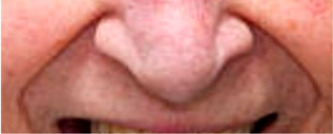
Cheek lines	Cheek	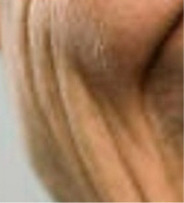
Marionette lines	Corner of mouth	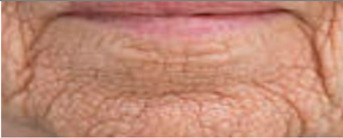

**Table 5:** Distribution of symptom training datasets.

Training datasets	500	1000	1500
Acne	250	500	750
Freckles	125	250	375
Wrinkles	125	250	375

**Table 6:** YOLOv3 Analysis of the training process (mAP = 0.5, Testing sets = 166).

Training	500	1000	1500
Iteration of model (×10k)	39	43	52
Best	55.50	52.78	57.52
Worst	45.09	44.35	50.47
Average	51.30	48.56	53.57

**Table 7:** YOLOv4 Analysis of the training process (mAP = 0.5, Testing sets = 166).

Training	500	1000	1500
Iteration of model (×10k)	88	115	100
Best	59.90	60.29	62.87
Worst	48.96	49.00	47.90
Average	55.84	54.96	57.83

**Table 8:** Mask R-CNN Face Detection Accuracy (mAP = 0.5, Testing sets = 160).

Training	100	250	500
Epoch (×100)	10	10	10
Accuracy	83.38	85.74	85.84

**Table 9:** Method Comparison (mAP = 0.5, Testing sets = 61).

Method\Training	500	1000	1500
YOLOv3	54.52	50.01	55.68
YOLOv4	58.74	56.98	56.29
Mask R-CNN + YOLOv3 (100 images for training)	50.03	47.43	56.53
Mask R-CNN + YOLOv3 (250 images for training)	50.38	46.54	53.70
Mask R-CNN + YOLOv3 (500 images for training)	55.02	52.39	58.13
Mask R-CNN + YOLOv4 (100 images for training)	55.97	57.64	53.68
Mask R-CNN + YOLOv4 (250 images for training)	55.10	56.48	53.19
Mask R-CNN + YOLOv4 (500 images for training)	57.73	60.38	59.75

## Data Availability

Not applicable.
